# Corrosion Behavior of Annealed 20MnCr5 Steel

**DOI:** 10.3390/ma18153566

**Published:** 2025-07-30

**Authors:** Dario Kvrgić, Lovro Liverić, Paweł Nuckowski, Sunčana Smokvina Hanza

**Affiliations:** 1 Faculty of Engineering, University of Rijeka, Vukovarska 58, 51000 Rijeka, Croatia; dario.kvrgic@riteh.uniri.hr (D.K.); suncana.smokvinahanza@riteh.uniri.hr (S.S.H.); 2 Faculty of Engineering, Juraj Dobrila University of Pula, Negrijeva 6, 52100 Pula, Croatia; 3 Materials Research Laboratory, Faculty of Mechanical Engineering, Silesian University of Technology, ul. Konarskiego 18a, 44-100 Gliwice, Poland; pawel.nuckowski@polsl.pl

**Keywords:** annealing heat treatment, microstructure, corrosion behavior, 20MnCr5 steel, mechanical properties

## Abstract

This study investigated the influence of various annealing treatments on the microstructure and corrosion behavior of 20MnCr5 steel in a 3.5% NaCl solution. A combination of microstructural analysis, hardness testing, and electrochemical techniques was used to comprehensively characterize each condition. To enhance data interpretability, a correlation analysis was performed and visualized through a correlation diagram, enabling statistical assessment of the relationships between grain features, phase distribution, mechanical properties, and corrosion indicators. The results demonstrated that corrosion resistance in 20MnCr5 steel is not governed by a single parameter but by the interplay between grain size, morphology, and phase balance. Excessive pearlite content or coarse, irregular grains were consistently associated with higher corrosion rates and lower electrochemical stability. In contrast, a moderate phase ratio and equiaxed grain structure, achieved through normalization, resulted in better corrosion resistance, confirmed by the highest polarization resistance and lowest corrosion current density values among all samples. Although increased grain refinement improved the hardness, it did not always correlate with a better corrosion performance, especially when morphological uniformity was lacking. This highlights the importance of balancing mechanical and corrosion properties through carefully controlled thermal processing.

## 1. Introduction

Heat treatment represents a key engineering method for optimizing the microstructure, and consequently the mechanical and corrosion properties of structural steels. Among the various heat treatment techniques, annealing holds a prominent position due to its ability to control phase balance, grain size and shape, and homogenize the material’s structure [1,2]. Depending on process parameters, such as temperature, holding time, and cooling rate, annealing can significantly influence the distribution of pearlite and ferrite as well as the presence of carbides or residual stresses, ultimately determining the material’s behavior in service conditions [3].

In industrial practice, particularly in the production of automotive and mechanical components, the ability to tailor microstructural features through thermal processing directly contributes to increased component reliability and service life. The impact of microstructure on corrosion performance has received growing attention, as it has been demonstrated that corrosion resistance is not solely governed by surface chemistry or alloy composition, but is also strongly influenced by features such as grain boundary density, phase morphology, and the distribution of inclusions or carbides.

Recent studies emphasize that microstructural tailoring via annealing, particularly the adjustment of ferrite-pearlite morphology, carbide precipitation, and grain boundary characteristics, can lead to improved surface passivation and lower corrosion rates in chloride-rich environments.

In [4], the authors investigated the effect of annealing on the microstructure and corrosion behavior of a low-alloy AISI 5140 steel in a 3.5% NaCl solution, comparing the as-received and annealed conditions. In [5], it was shown that spheroidized steel, especially when combined with Cu microalloying, significantly improved the corrosion resistance by reducing micro-galvanic coupling between ferrite and cementite in a chloride-containing environment. In [6], the authors demonstrated that spheroidized steel exhibited a slower corrosion rate during long-term immersion, which was attributed to the weaker micro-galvanic effect caused by the reduced accumulation of cathodic cementite.

In [7], a high-strength low-alloy (HSLA) steel was examined under various heat treatment conditions. The study found that a high-temperature tempered martensitic microstructure provided superior corrosion resistance (evaluated via 5% NaCl salt-spray tests) compared with other conditions, highlighting the role of tempering, particularly stress-relief and carbide precipitation, in improving corrosion performance.

In [8], four plain-carbon steels (with 0% to ~100% pearlite) were studied under various annealing and normalizing conditions. The findings showed that pearlite refinement through normalizing (air cooling) significantly lowered the corrosion rates in 3.5% NaCl by reducing interlamellar spacing and pearlite colony size, whereas coarse pearlite produced via slow furnace cooling was more susceptible to galvanic corrosion between ferrite and cementite.

20MnCr5 is a low-alloy case-hardening steel that is frequently used for gears, shafts, and bearing parts. In industrial practice, it is routinely subjected to a sequence of thermal processes: most commonly carburizing at 900–950 °C, followed by quenching and tempering at low temperatures (200–220 °C) to produce a hard, wear-resistant surface; normalization (880–900 °C, air cooling) or full annealing (830–860 °C, slow furnace cooling) to refine equiaxed grains and relieve stresses; spheroidizing annealing (700–740 °C, prolonged holding and slow cooling) to improve machinability; and occasional stress relieving treatments (650–700 °C) to remove residual stresses from previous machining. These heat treatments allow for the independent tuning of surface hardness, core toughness, and corrosion resistance. Due to its service in high-contact and high-load conditions, the precise control of both the surface and core properties is critical for reliable performance and a long service life.

Recent studies have shown that specific annealing and normalizing treatments can induce significant microstructural changes in 20MnCr5 including the development of refined equiaxed grain structures, the spheroidization of carbides, and adjustment in the ferrite-pearlite phase balance [9]. Such microstructural tailoring has been shown to enhance surface passivation and significantly reduce the corrosion rates in aggressive chloride-containing environments such as 3.5% NaCl solutions [10].

Moreover, carburizing and subsequent tempering treatments influence the distribution and morphology of carbides, which in turn affect both the mechanical and electrochemical behavior of the steel [11]. Carburizing at optimized temperatures (around 900–950 °C) refines the surface microstructure by promoting a fine martensitic grain structure and reducing retained austenite, improving hardness and wear resistance without compromising corrosion performance [12]. Tempering treatments (typically low-temperature tempers around 200 °C for case-hardened 20MnCr5) relieve residual stresses from quenching and precipitate finely dispersed carbides, which benefits both corrosion resistance and toughness. The tempering-induced carbide precipitation is distributed in a uniform, globular form [11], and the reduction in internal stresses helps mitigate crack initiation sites. These changes lead to improved impact toughness and fatigue performance of the hardened case without sacrificing its corrosion behavior [9].

Taken together, these findings highlight that controlled heat treatment strategies aimed at microstructural engineering are essential to optimize the multi-functional performance of 20MnCr5 steel components in demanding industrial applications. By tailoring microstructure through processes such as annealing, normalizing, carburizing, and tempering, engineers can achieve a favorable combination of high surface hardness, core toughness, and enhanced corrosion resistance [10,12,13,14,15,16,17,18,19], ensuring a long service life for 20MnCr5-based parts in aggressive service conditions.

Modern electrochemical characterization methods enable a detailed analysis of the influence of certain heat treatment processes on corrosion behavior. Correlation analyses between microstructural features and electrochemical parameters, such as open circuit potential (OCP), linear polarization resistance (LPR), electrochemical impedance spectroscopy (EIS), and potentiodynamic polarization (PDP), are increasingly employed as powerful tools to relate the grain structure, phase balance, and residual stress state to corrosion performance in chloride-containing environments [4,5,6,7,8,10,12,13,14,15,16,17,18,19].

This study investigated the effect of different annealing regimes on the microstructure and corrosion resistance of 20MnCr5 steel in a 3.5% NaCl solution. The applied annealing treatments included normalizing, stress relieving, soft annealing, full annealing, spheroidizing annealing, and transformation annealing. Normalizing (N) was performed to refine the austenitic grain size, improve machinability, and reduce distortion after subsequent heat treatment [3]. Stress relieving (+SR) was used to reduce residual stresses induced by prior machining or deformation without significantly altering the microstructure. Soft annealing (A) aims to improve the machinability by reducing hardness and homogenizing the structure. Full annealing (FA) was applied to achieve the maximum softness and ductility by producing a coarse, equiaxed ferrite-pearlite microstructure. Spheroidizing annealing (AC) promotes the formation of globular carbides to enhance formability and improve machinability, especially before cold forming. Transformation annealing (+FP) was used to obtain a uniform and fine microstructure by transforming the phases through controlled heating and cooling cycles.

The findings provide valuable insights into how annealing parameters can be tailored to optimize both the structural integrity and durability in corrosive environments. Such knowledge is particularly relevant for the design of components exposed to marine or industrial chloride conditions, where microstructural engineering plays a critical role in long-term performance and material reliability.

## 2. Materials and Methods

### 2.1. Material

The material used in this study was case-hardening 20MnCr5 steel, characterized by a low carbon content and good hardenability. It was supplied in the form of factory-produced bars with a diameter of 16 mm.

The average chemical composition of the 20MnCr5 steel was determined using a LECO GDS 500 (LECO Corporation, St. Joseph, MI, USA) a optical glow discharge spectrometer, based on the mean values obtained from five individual measurements. The detailed chemical composition is shown in Table 1.

### 2.2. Treatment

From the original 20MnCr5 steel bar, six disc-shaped samples with a thickness of 4 mm were cut for the purpose of thermal processing. Each sample was subjected to a different annealing heat treatment in order to obtain a range of microstructures and corresponding properties. The applied annealing treatments and their parameters are listed in Table 2.

Specifically, the samples underwent the following treatments: Sample 1—normalizing (N), Sample 2—stress relieving (+SR), Sample 3—soft annealing (A), Sample 4—full annealing (FA), Sample 5—spheroidizing annealing (AC), and Sample 6—transformation annealing (+FP).

The selection of heat treatment parameters (such as heating temperature, holding time, and cooling rate) was based on the technical datasheet for 20MnCr5 steel, ensuring that each regime was industrially relevant and appropriate for achieving the intended microstructural modifications.

The applied annealing heat treatments were designed to produce a range of microstructures in 20MnCr5 steel by controlling the heating temperatures, holding times, and cooling rates. According to the technical datasheet, the *A*_c1_ and *A*_c3_ transformation temperatures for 20MnCr5 steel are approximately 730 °C and 830 °C, respectively. *A*_c1_ is the lower critical temperature, marking the onset of transformation from pearlite to austenite, while *A*_c3_ is the upper critical temperature, at which the transformation from ferrite to austenite is complete, resulting in a fully austenitic structure. The temperature range between *A*_c1_ and *A*_c3_ defines the intercritical region, where partial transformation to austenite occurs.

These critical transformation temperatures served as the basis for determining the thermal regimes. Normalization, full annealing, and transformation annealing involved heating the samples above the *A*_c3_ temperature to achieve complete austenitization. In contrast, stress relieving, soft annealing, and spheroidizing annealing were carried out at subcritical temperatures, typically below the *A*_c1_ threshold.

### 2.3. Hardness Measurements

The hardness of the samples after heat treatment was measured to verify the effectiveness and correctness of the applied thermal processes. Since each annealing treatment was expected to result in a specific microstructural transformation—reflected in the characteristic hardness values—the measured hardness was compared with the expected values specified in the steel’s technical datasheet. This comparison served as an indirect confirmation that the targeted microstructures had been achieved.

The measurements were performed using a ZHU 187.5 (Zwick Roell Group, Ulm, Germany) universal hardness tester, applying the Rockwell B (HRB) scale, with a steel carbide ball indenter of 1.588 mm in diameter. Consistency between the measured and expected hardness values indicated that the selected heat treatment parameters (temperature, holding time, and cooling rate) were appropriately executed for each sample.

### 2.4. Microstructural and Phase Analysis

The microstructural characterization of 20MnCr5 steel was performed both in the as-received normalized condition and after the applied annealing heat treatments. Metallographic preparation was carried out using standard grinding and polishing procedures on a STRUERS LABOPOL system (Struers; Ballerup, Denmark). The initial grinding was performed on a resin-bonded MD-Piano 220 diamond disc (Struers), followed by polishing on an MD-Allegro composite disc (Struers) with diamond suspensions of decreasing particle size. The final polishing steps were conducted using an MD-Dac cloth (Struers) with DiaPro Dac (Struers) 3 µm and 1 µm diamond suspensions, and an MD-Chem cloth (Struers) with an OP-U NonDry colloidal silica suspension (Struers).

Etching was performed using a 3% Nital solution for 10–20 s to reveal the microstructural features. The samples were examined using an optical microscope (BX-51, Olympus Corporation, Tokyo, Japan) at various magnifications. Phase distribution analysis and quantification of the pearlite and ferrite phases were performed using Olympus Stream Essentials software (Olympus BX51 microscope). Grain size and morphology were further analyzed using MATLAB® (R2022a, MathWorks, Natick, MA, USA) with custom image analysis scripts, enabling the extraction of parameters such as maximum and minimum grain diameter, equivalent diameter, area, perimeter, and circularity for both the ferrite and pearlite phases.

In addition, X-ray diffraction (XRD) analysis was performed on all samples using a PANalytical X’Pert Pro MPD diffractometer (Panalytical B.V. (currently: Malvern Panalytical Ltd.), Almelo, The Netherlands) equipped with a copper anode X-ray tube (Kα Cu = 0.154 nm) and a Ni filter to suppress Kβ radiation to confirm the phase composition and assess possible differences in cementite distribution related to the applied cooling rates. The XRD patterns were collected in the 2θ range from 10° to 110° with a recording step of 0.05°, and the identified peaks were indexed according to the standard diffraction data for α-Fe and Fe_3_C phases.

### 2.5. Electrochemical Testing

Electrochemical measurements were carried out using a conventional three-electrode configuration connected to an Interface 1010E potentiostat (Gamry Instruments, Warminster, PA, USA) [20,21]. As the electrolyte, a 3.5 wt.% aqueous NaCl solution was used and maintained at ambient temperature [22,23]. The investigated sample served as the working electrode, while a saturated Ag/AgCl electrode and a graphite rod were employed as the reference and counter electrodes, respectively. Prior to testing, all samples were mechanically polished with silicon carbide (SiC) abrasive papers up to 4000 grit to achieve a consistent and smooth surface finish.

To monitor system stabilization, the open-circuit potential (OCP) was continuously recorded over a 60-min period [24]. Following this stabilization phase, linear polarization resistance (LPR) measurements were conducted by applying a ±20 mV potential shift relative to the stabilized OCP. The polarization resistance (*R*_p_) was determined from the linear region of the current–potential response [24,25].

Electrochemical impedance spectroscopy (EIS) was utilized to assess corrosion mechanisms at the electrode–electrolyte interface. EIS spectra were collected at the OCP within the frequency range of 100 kHz to 0.01 Hz, applying a 10 mV sinusoidal signal [26]. The resulting data were interpreted by fitting to an equivalent electrical circuit to characterize the charge transfer and diffusion-related processes.

Additionally, potentiodynamic polarization tests were performed to study the corrosion kinetics in more detail. The potential scan ranged from –0.6 V to +2.5 V versus OCP, at a sweep rate of 2 mV/s. Key parameters including the corrosion potential (Ecorr), corrosion current density (icorr), and calculated corrosion rates were obtained using the Tafel extrapolation technique [24,27].

## 3. Results

### 3.1. Microstructural Characterization

#### 3.1.1. Microstructural Features

**Figure 1:**
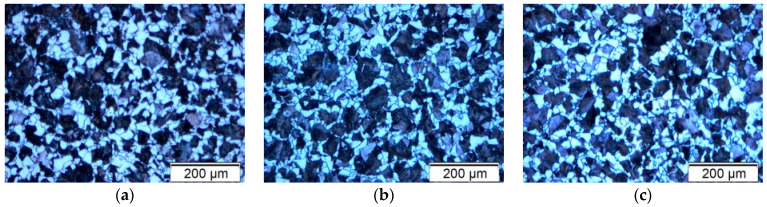

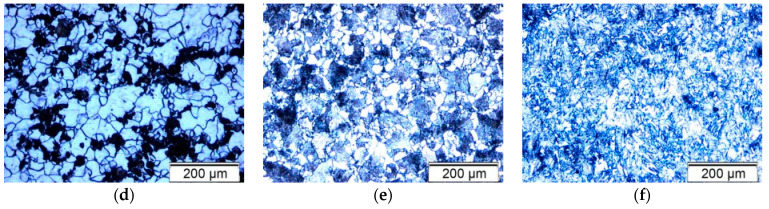
Optical microscope micrographs of the annealed samples: (**a**) Sample 1 (N), (**b**) Sample 2 (+SR), (**c**) Sample 3 (A), (**d**) Sample 4 (FA), (**e**) Sample 5 (AC), and (**f**) Sample 6 (+FP).

Figure 1 shows the microstructures of the 20MnCr5 steel after the various annealing processes presented in Table 2, which were photographed with an optical microscope at 200× magnification. The heterogeneous microstructure of the normalized 20MnCr5 steel, consisting of ferrite crystals embedded in pearlite colonies, is shown in Figure 1a. The microstructure showed clearly distinguishable pearlite and ferrite phases, which were later confirmed by the XRD analysis. The microstructure consisted predominantly of ferrite grains interspersed with distinct pearlite colonies, indicating a heterogeneous structure [13,15,16,17,18].

In the microscopic images, the pearlite phase appeared darker, while the ferrite phase appeared lighter. The ratio of pearlite to ferrite depends primarily on the chemical composition (especially the carbon content) and the heat treatment parameters, with the cooling rate playing a critical role [15,16,17,18]. The overall microstructures observed were generally similar. In all micrographs, pearlite was randomly distributed within the ferrite matrix, although the grain size varied. In some cases, especially in Figure 1d,e, a dominance of the ferrite phase over pearlite could be seen. Furthermore, the influence of different cooling rates on the diffusion-dependent nucleation and growth of spherical cementite particles in pearlite could clearly be seen when comparing the microstructures in Figure 1a,e,f [17,18]. In addition, the beginning of the second stage of spheroidization could be seen in Sample 5 (Figure 1e), which was characterized by the dissolution of fine cementite particles and the growth of coarser particles.

**Figure 2:**
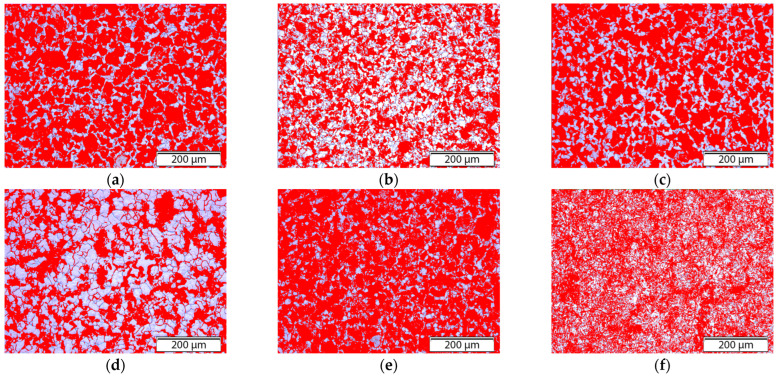
Optical microscope micrographs of the annealed samples after pearlite phase analysis: (**a**) Sample 1 (N), (**b**) Sample 2 (+SR), (**c**) Sample 3 (A), (**d**) Sample 4 (FA), (**e**) Sample 5 (AC), and (**f**) Sample 6 (+FP).

Figure 2 shows the microstructures of 20MnCr5 steel analyzed using Olympus BX51 software after various annealing processes, observed with an optical microscope at 100× magnification. The samples exhibited similar grain boundary structures but differed noticeably in phase distribution and grain size, as summarized in Table 3.

The spheroidized sample (Sample 5, AC) exhibited the highest proportion of pearlite within the microstructure, while the full annealed sample (Sample 4, FA) had the lowest pearlite content, as shown in Table 3. These differences in grain size and phase distribution were closely related to the annealing parameters (Table 2), particularly the heating and cooling rates, which influence the grain growth and phase transformation during the thermal cycle [13,14,15,16,17,18].

#### 3.1.2. Grain Size and Morphology Analysis

**Figure 3:**
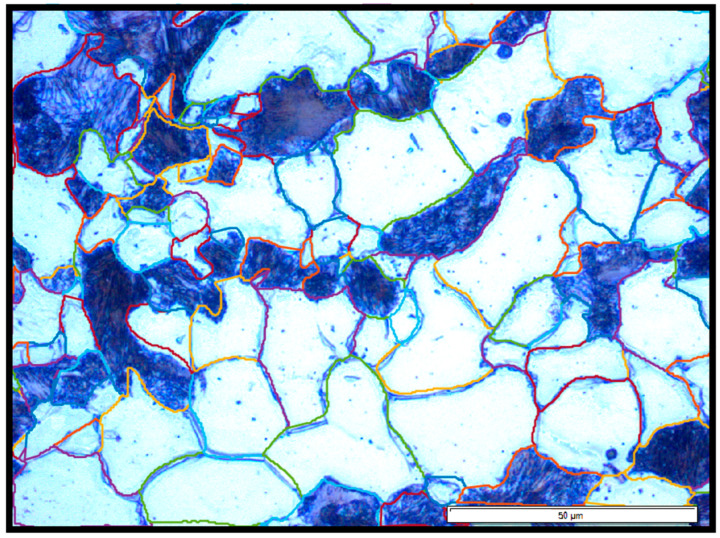
Example of the grain size analysis process for Sample 4 (FA), following the same procedure applied to all other samples. Grain boundaries were detected and highlighted using different colors to distinguish grain orientations and segmentation results: red lines indicate primary grain boundaries, yellow lines represent sub-grain structures or low-angle boundaries, and green lines mark secondary segmentation corrections.

To quantify the grain morphology and assess the effects of various heat treatments on the steel microstructure, grain size analysis was performed on etched samples of 20MnCr5 steel. Representative micrographs of the analyzed microstructures are shown in Figure 3. The analysis was conducted using a custom-developed MATLAB script designed to extract geometrical and morphological parameters from optical micrographs.

The key parameters measured included the maximum diameter (D_max_), minimum diameter (D_min_), equivalent diameter (Equiv. Diameter), area, and circularity. These parameters were evaluated for each identified grain in both the ferritic (white grains) and pearlitic (dark grains) phases, as summarized in Table 4.

The maximum diameter (D_max_) represents the largest linear distance across a grain, while the minimum diameter (D_min_) represents the shortest linear distance across the same grain. The equivalent diameter corresponds to the diameter of a circle with the same area as the grain, facilitating comparisons with ideal geometries. Grain area refers to the projected surface area of the grain, while circularity is a dimensionless parameter describing how closely a grain’s shape resembles a perfect circle. Higher circularity values indicate more equiaxed grains, whereas lower values suggest elongated or irregular morphologies.

Table 4 presents the grain size analysis results for the investigated 20MnCr5 steel samples, distinguishing between the ferritic (white grains) and pearlitic (dark grains) phases. Among the ferritic grains, Sample 4 (FA) exhibited the largest size, with a D_max_ of 41.49 µm and a corresponding area of 888.27 µm^2^. In contrast, Sample 6 (+FP) showed the smallest ferrite grains, with the lowest equivalent diameter (12.74 µm) and grain area (150.67 µm^2^). The finer ferrite microstructure in Sample 6 suggests rapid cooling during processing, which may have contributed to increased mechanical strength, as seen in Table 5.

A similar trend was observed in the pearlitic phase. The coarsest pearlite grains were recorded in Sample 3 (A), with D_max_ reaching 61.11 µm and an area of 1572.21 µm^2^. On the other hand, the finest pearlite grains again appeared in Sample 6 (+FP), characterized by a D_max_ of 33.20 µm and an area of just 231.66 µm^2^. These results consistently indicate microstructural refinement across both phases in Sample 6 (+FP).

Circularity analysis reinforced these findings. Samples 4 (FA) and 5 (AC) exhibited relatively high circularity values in the pearlitic phase (0.57 and 0.55, respectively), indicative more equiaxed grains, likely formed during full annealing or spheroidization. Conversely, Sample 6 (+FP) demonstrated the lowest circularity in both ferrite (0.42) and pearlite (0.38), suggesting more elongated or irregular grain shapes, potentially resulting from directional solidification or fast transformation kinetics during cooling.

#### 3.1.3. Phase Investigation

**Figure 4:**
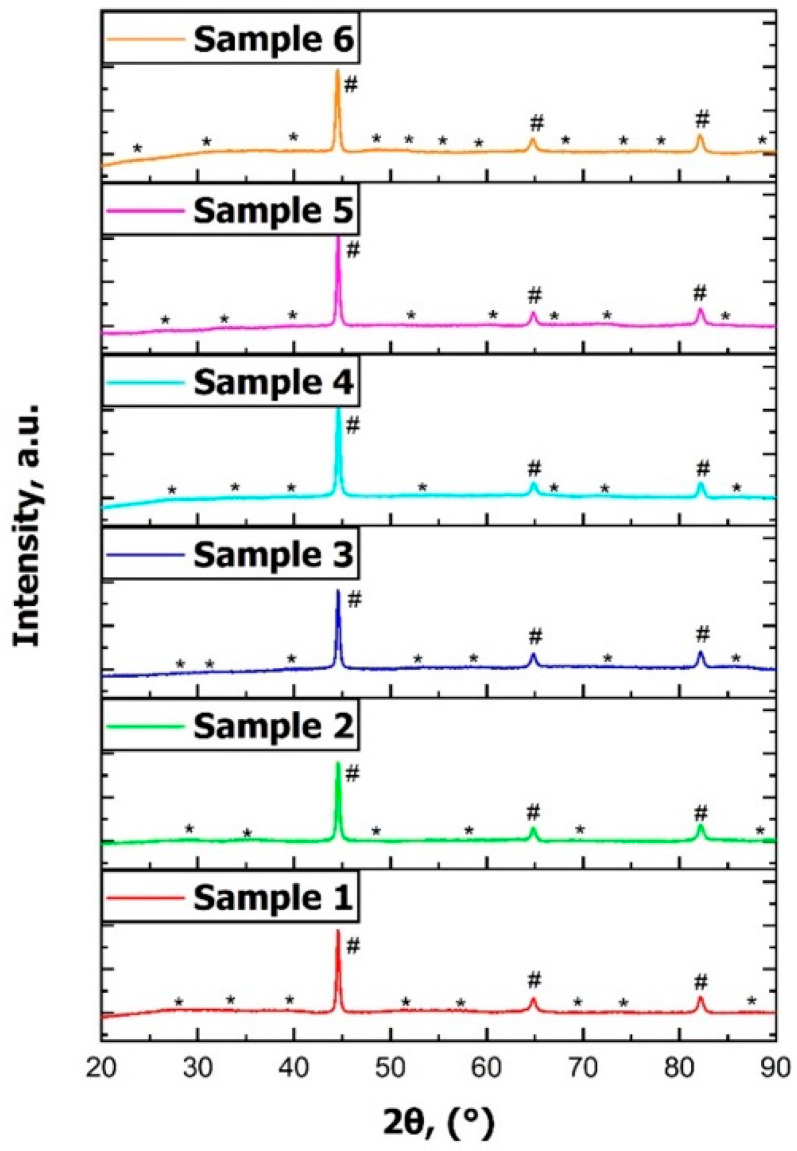
XRD diffractogram of samples: Sample 1 (N), Sample 2 (+SR), Sample 3 (A), Sample 4 (FA), Sample 5 (AC), and Sample 6 (+FP), # represents α iron (α-Fe), * represents cementite (Fe_3_C).

The diffractograms display the relative intensities of the phases present in each sample investigated. The indexed peaks correspond to the following phases: # represents α iron (α-Fe), while * represents cementite (Fe_3_C). All samples exhibited the α-Fe phase, also known as ferrite, along with cementite (Fe_3_C), which was expected and is a normal finding in these grades of steels [15,19]. The XRD patterns are presented in terms of reticular distances (2θ). As seen in Figure 4, the phase densities appeared relatively similar across the samples. However, differences in the cementite distribution could be seen, likely due to variations in the cooling rates during heat treatment. The changes in cementite peak intensities, especially in certain 2θ regions, revealed how the cooling rates affect the microstructure.

#### 3.1.4. Hardness Measurements

The hardness values listed in Table 5 represent the average of five measurements with the standard deviation. The samples were polished to remove any potential surface oxide or contaminants prior to hardness testing.

**Table 5:** Hardness Rockwell B measurements of the samples.

Hardness Measurements	
Sample	Average
1	90 ± 3
2	87.2 ± 3
3	87 ± 3
4	81.4 ± 3
5	82 ± 3
6	98.4 ± 3

The hardness values obtained after annealing are consistent with the data reported in the experimental literature [14,15,16,17,18,19]. The results show that Samples 1, 2, and 3 had similar hardness values, while Samples 4 and 5 exhibited significantly lower hardness, and Sample 6 showed a significantly higher hardness.

### 3.2. Electrochemical Investigations

#### 3.2.1. Open Circuit Potential (OCP)

**Figure 5:**
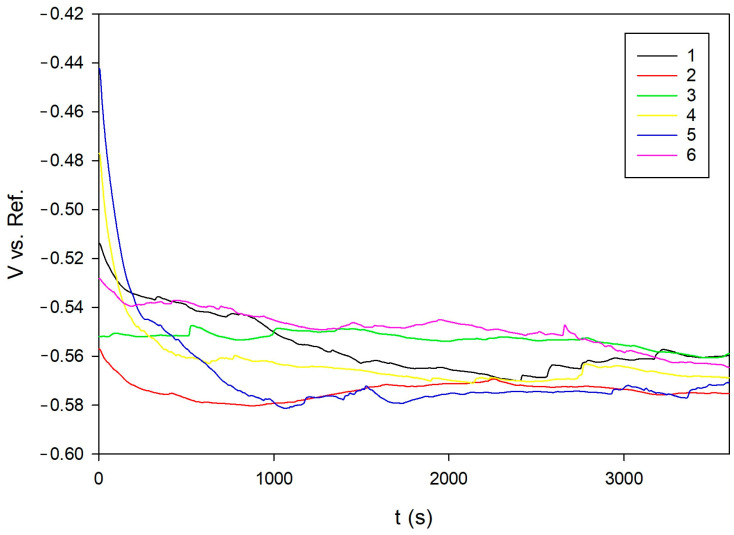
OCP evolution of the 20MoCr5 steel samples in 3.5 wt.% NaCl solution over time.

All investigated specimens exhibited negative open circuit potential (OCP) values, ranging from –0.5594 V (Sample 1) to –0.5752 V (Sample 2) vs. the Ag/AgCl reference electrode, indicating the electrochemical activity of 20MnCr5 steel in chloride-containing environments, which can be seen in Figure 5 [10,28]. Although the observed variations between samples were relatively small, they remain relevant for assessing the initial stability of the surface.

Sample 1 showed the most positive potential, suggesting a more favorable surface chemistry and reduced anodic activity [20,21,28]. In contrast, Sample 2 exhibited the most negative potential, implying a higher tendency for metal dissolution. The remaining samples clustered within a narrow range (–0.5605 V to –0.5716 V), with minor differences likely attributed to microstructural variations and surface heterogeneity induced by heat treatment [29].

Overall, the negative potential values across all samples were consistent with the thermodynamic instability of 20MoCr5 steel under chloride exposure, and they confirm the thermodynamic tendency for active corrosion behavior under chloride exposure [20,21,28].

#### 3.2.2. Linear Polarization Resistance (LPR)

The measured polarization resistance (*R*_p_) values ranged from 60.70 Ω (sample 5) to 167.30 Ω (sample 1), as seen on Table 6, indicating variations in the initial corrosion resistance. Given that *R*_p_ inversely reflects the corrosion rate and is often employed to identify materials suitable for further electrochemical analysis, these variations are considered meaningful [24,25].

The highest *R*_p_ of Sample 1 suggests a lower corrosion rate, while Samples 5 and 6, with the lowest *R*_p_, likely suffered from surface defects, consistent with their more negative OCP values [24,25].

Sample 4, despite its relatively positive OCP, exhibited only a moderate *R*_p_ (105.30 Ω), which may reflect microstructural inhomogeneities or locally active surface regions not captured by OCP alone [21].

These results indicate that OCP alone is insufficient to assess corrosion resistance, particularly in chloride-containing systems. The combined evaluation of OCP and *R*_p_ provides a more reliable assessment of early-stage electrochemical stability [24,25].

#### 3.2.3. Electrochemical Impedance Spectroscopy (EIS)

Electrochemical impedance spectroscopy was performed on six 20MoCr5 steel samples subjected to various heat treatments to evaluate their corrosion behavior in 3.5 wt.% NaCl. Impedance spectra were interpreted using the equivalent circuit model *R*_s_–[(CPE_f_‖(*R*_f—_*CPE_dl_‖(R_ct—_W*))], capturing sequential processes from the electrolyte to the metal substrate [30].

**Figure 6:**
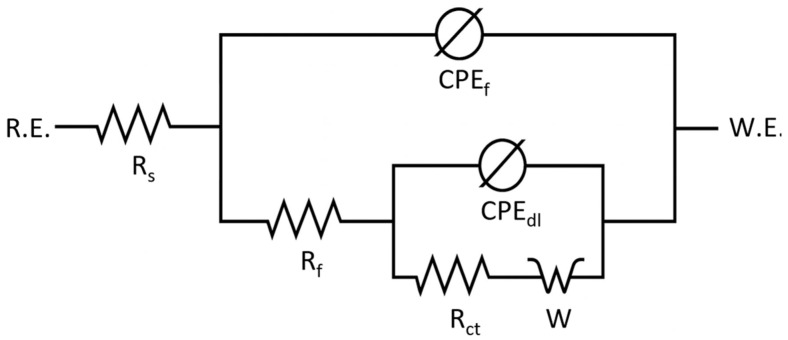
Equivalent electrical circuit used to fit the EIS data of the 20MoCr5 steel samples in 3.5 wt.% NaCl solution.

In this model (Figure 6), *R_s_* represents the solution resistance, *CPE_f_* models the non-ideal capacitive behavior of the porous surface layer, *R_f_* is the resistance of that layer, *CPE_dl_* corresponds to the double-layer capacitance at the metal interface, *R_ct_* is the charge transfer resistance, and *W* represents the Warburg impedance related to ion diffusion. This structured arrangement enables the physical interpretation of the complex impedance response, particularly relevant in localized corrosion scenarios [30,31].

Nyquist plots for all samples exhibited two partially overlapping semicircles, corresponding to the porous surface layer *CPE_f_*‖*R_f_*, while the mid-to-low-frequency arc reflected the metal–electrolyte interface, described by *CPE_dl_*‖*R_ct_*. A tail appearing in the low-frequency region indicated diffusion-limited transport, modeled by the Warburg element *W*, with sample-dependent variations in both slope and curvature [30,31].

**Figure 7:**
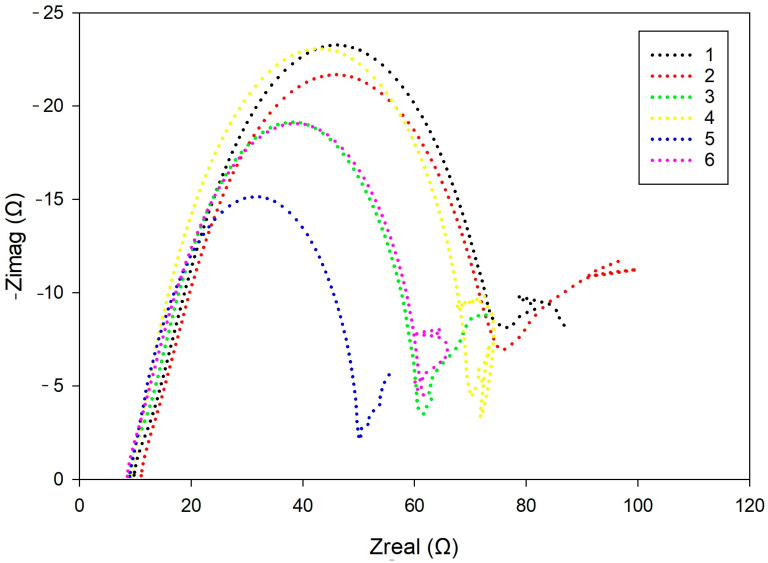
Nyquist plots for all samples. The two semicircular features represent electrochemical responses of the porous corrosion layer and the substrate interface.

Nyquist plots for all samples, as shown in Figure 7, exhibited a two-time-constant behavior, with two partially overlapping semicircular features corresponding to distinct electrochemical processes. The first, smaller arc, typically more depressed and incomplete, was attributed to the *CPE_f_*‖*R_f_* element, associated with the porous, oxidized corrosion layer. Its non-ideal shape (n < 1) suggests surface inhomogeneities and limited protective properties [30,31,32].

The second, larger arc reflects the *CPE_dl_*‖*R_ct_* response, associated with the double-layer capacitance and charge transfer resistance at the metal–electrolyte interface beneath the porous corrosion layer. This feature represents the kinetics of the electrochemical reactions occurring on the exposed metal surface. Variations in its shape and magnitude among samples indicate differences in interfacial reactivity and progression of the corrosion process.

In all samples, a characteristic tail was observed at the end of the impedance curve, indicating diffusion-controlled behavior. Differences in its slope and shape point to variations in the morphology and compactness of the surface corrosion products.

**Figure 8:**
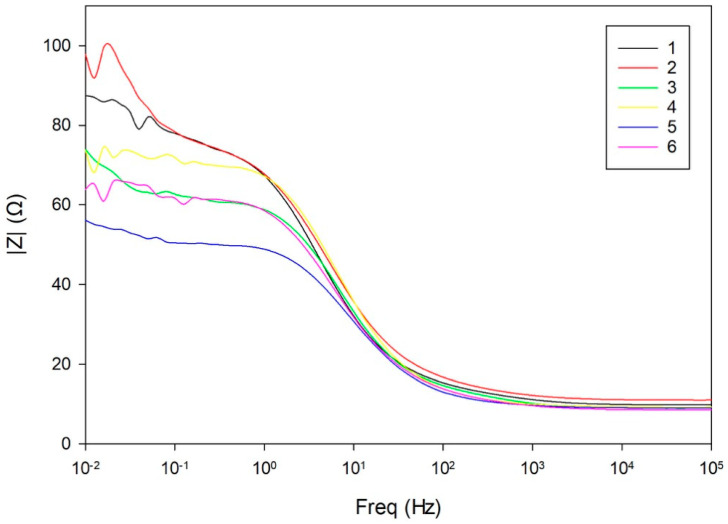
Bode plots of impedance magnitude ∣Z∣ as a function of frequency for all samples.

**Figure 9:**
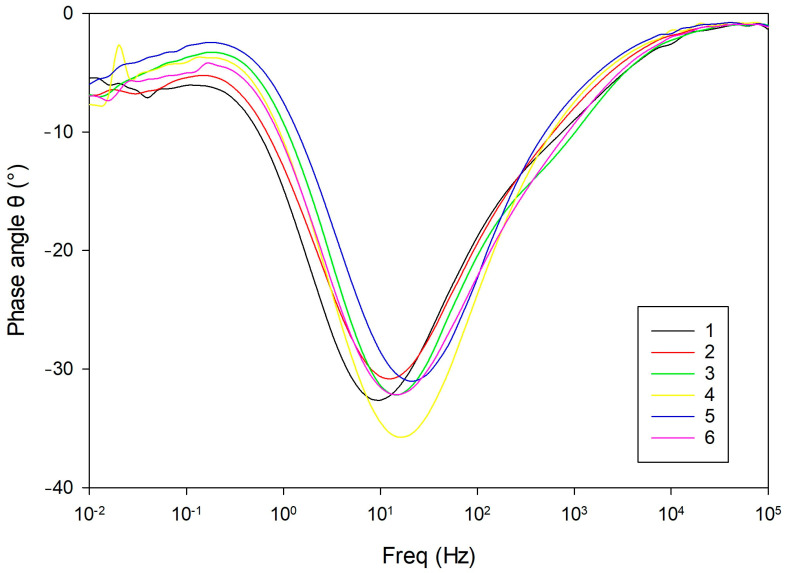
Phase angle (θ) versus frequency plots for all samples, revealing two overlapping relaxation processes.

Bode plots of the impedance magnitude (Figure 8) and phase angle (Figure 9) displayed a typical three-segment response. At high frequencies, all samples showed similar |*Z*| values and a flat region, indicating dominance of the solution resistance (*R_s_*). This was followed by a ~45° slope in the mid-frequency range, associated with capacitive behavior of the porous surface layer *CPE_f_*‖*R_f_* [30,31].

Toward lower frequencies, the impedance curves diverged, transitioning into a near-horizontal region indicative of diffusion-limited processes and increased system resistance. Sample-dependent differences in slope and smoothness suggest variations in corrosion product morphology and surface integrity.

Phase angle plots (Figure 9) revealed two overlapping relaxation processes. An initial plateau near 0° confirmed ohmic behavior from the electrolyte *R_s_*. As the frequency decreased, the phase angle dropped to a minima between –29° and –36°, reflecting capacitive dispersion of the porous surface layer *CPE_f_*‖*R_f_*. At the lowest frequencies, the phase angles rose again toward ~–5°, indicating the influence of the metal–electrolyte interface *CPE_dl_*‖*R_ct_* and the diffusion-related limitations described by the Warburg element. Divergences in this region suggest interfacial heterogeneity or kinetic instability in some samples.

The obtained results indicate that stable, protective oxide layers did not form on the surface of the tested samples. Instead, the observed behavior was characteristic of localized corrosion, evidenced by the low resistance of the corroded layer (*R*_f_) and high constant phase element (CPE_f_) values. The exponents n_1_ and n_2_, corresponding to the CPE_dl_ and CPE_f_ elements, respectively, are dimensionless parameters that describe the deviation of each CPE from ideal capacitive behavior. Values close to 1 indicate nearly ideal capacitance, while lower values (n < 1) reflect surface heterogeneity, porosity, or roughness, which are consistent with the observed dispersion in the impedance response. Such dispersion (n < 1) highlights the non-ideal electrochemical behavior of the tested surfaces [31,33]. These features are indicative of porous, rough, and electrochemically heterogeneous surfaces (Table 7). Warburg impedance was present in all samples, with its intensity varying depending on the degree of corrosion product accumulation and the diffusional permeability of the surface layers.

As shown in Table 7, Samples 1 and 2 exhibited a relatively high charge transfer resistance (*R*_ct_), but low surface layer resistance *R_f_*, suggesting early-stage degradation. Sample 4 showed high capacitance and strong dispersion, likely resulting from pronounced surface roughness and uneven corrosion. Sample 5, characterized by the lowest *R_ct_* and the highest Warburg impedance, exhibited the most intense corrosion activity, dominated by diffusion control. In contrast, Sample 2 had the highest *R_f_* value, indicating partial surface passivation. However, this was accompanied by significant heterogeneity, suggesting the presence of isolated passive zones rather than a continuous protective film.

#### 3.2.4. Potentiodynamic Polarization (PDP)

Potentiodynamic polarization measurements were performed in 3.5 wt.% NaCl to determine the corrosion potential (*E*_corr_), corrosion current density (*I*_corr_), and corrosion rate (CR), following standard procedures described in ASTM G59 [25] and the manufacturer’s guidelines [24]. All tests were conducted at a scan rate of 2 mV/s over a wide anodic and cathodic potential range relative to the OCP. The results are summarized in Table 8.

**Figure 10:**
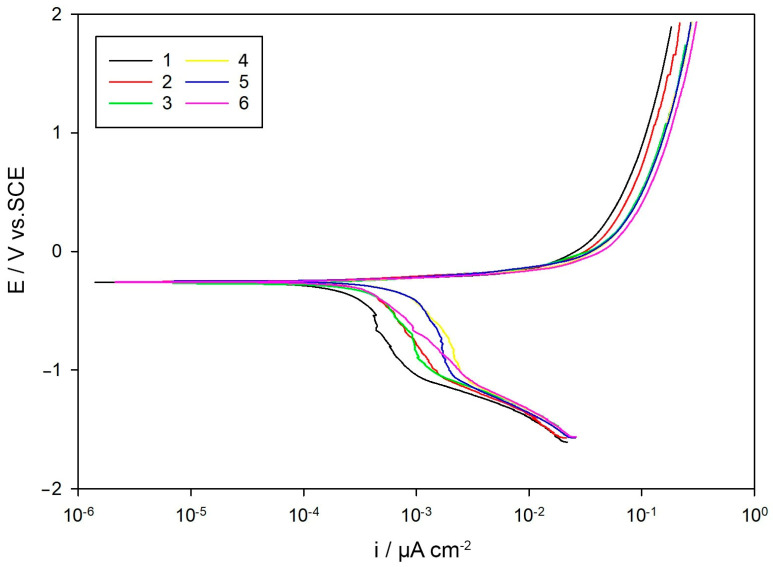
Potentiodynamic polarization curves of the investigated samples in 3.5 wt.% NaCl solution.

The polarization curves exhibited similar anodic behavior across all samples, visible in Figure 10, indicating a common active dissolution mechanism without the formation of stable passive films, as typically observed for low-alloy steels in chloride environments. Differences were more evident in the cathodic region, particularly near –1 V, where variations in slope suggest differences in cathodic reaction kinetics, likely influenced by the surface condition, defect density, and local microstructure [21,30].

Corrosion potentials ranged narrowly from –250.3 mV (sample 5) to –269.3 mV (sample 3), while the corrosion current densities varied significantly. The lowest *I*_corr_ was recorded for Sample 1 (90.19 µA/cm^2^), corresponding to a corrosion rate of 2.104 mm/year, whereas the highest values were observed for Samples 4 and 5, reaching 622.40 µA/cm^2^ and 486.90 µA/cm^2^, respectively.

The elevated *I*_corr_ values of Samples 4 and 5 suggest accelerated corrosion kinetics, likely due to increased microstructural heterogeneity or compromised surface layers caused by heat treatment, as previously reported for similar steel systems [10,29]. In contrast, Sample 1 exhibited the lowest corrosion rate, consistent with its low *I*_corr_ and highest *R*_p_ from the LPR analysis, supporting the established correlation between these electrochemical indicators [25,27]. These findings confirm that the corrosion resistance of 20MoCr5 steel is highly sensitive to heat treatment-induced microstructural changes, where even minor differences can lead to different corrosion behaviors [10,29].

## 4. Discussion

To better understand the corrosion behavior of annealed 20MnCr5 steel, a comprehensive approach was adopted that integrated microstructural characterization with multi-method electrochemical analysis [28]. While individual measurements such as open circuit potential (OCP), polarization resistance (LPR), impedance spectra (EIS), and potentiodynamic polarization (PDP) provide insights into specific aspects of corrosion mechanisms, a broader perspective was needed to identify interdependencies across the structural and electrochemical parameters.

**Figure 11:**
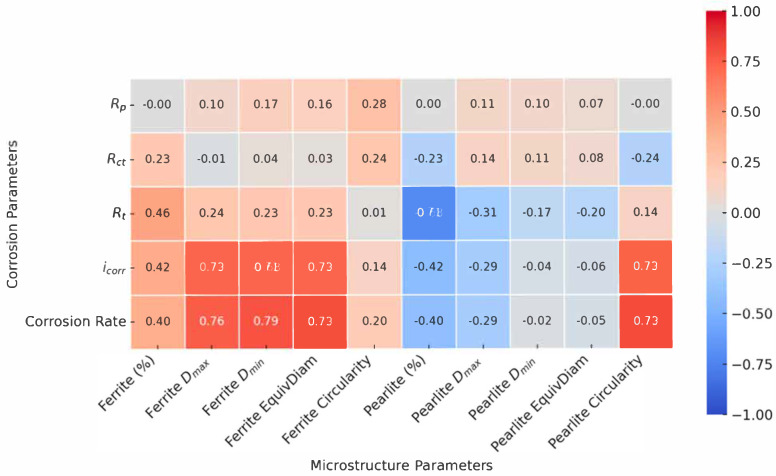
Correlation diagram of the Pearson correlation coefficients between the electrochemical, microstructural, and phase-structure parameters of annealed 20MnCr5 steel: red indicates strong positive correlation; blue, strong negative correlation.

Therefore, a correlation analysis was conducted to statistically link microstructural features (grain size, morphology, phase ratios), mechanical properties (hardness), and electrochemical responses (*R*_p_, *I*_corr_, *R*_1_, *R*_2_, corrosion rate, etc.) [34,35]. The resulting correlations were visualized using a correlation diagram (Figure 11), allowing for rapid identification of the strongest positive and negative relationships. This approach enabled an objective, data-driven interpretation of which microstructural characteristics most influenced the corrosion resistance, and provides a solid foundation for the discussion that follows.

In the revised analysis, the correlation diagram was adjusted to display only the relationships between the corrosion parameters and microstructural characteristics. Correlations within the same parameter groups (corrosion–corrosion or microstructure–microstructure) were excluded. The final diagram presents the corrosion properties on the y-axis and microstructural parameters on the x-axis, enabling a clearer interpretation of how microstructural features influence corrosion behavior (Figure 11). Strong positive correlations (deep red) and strong negative correlations (deep blue) highlight statistically significant relationships. The correlation analysis was conducted using the Pearson correlation coefficient. Positive correlation values (r > 0) indicate that higher concentrations of a given element are associated with increased electrochemical parameter values, whereas negative values (r < 0) suggest an inverse relationship. Correlation strength was interpreted as follows: weak (|r| < 0.5), moderate (0.5 ≤ |r| < 0.8), and strong (|r| ≥ 0.8) [35].

Based on the observed patterns, several key microstructural factors emerged as critical contributors to corrosion behavior, particularly the ferrite grain area, pearlite D_max_, and grain circularity.

The correlation map revealed a moderately strong positive correlation between the corrosion rate and pearlite content, especially when accompanied by a high pearlite D_max_ and low circularity. For instance, Sample 5 (AC), which had the highest pearlite content (71.53%) and large, irregular pearlitic grains, exhibited elevated corrosion current density and low substrate resistance. These results confirm that pearlite, although mechanically beneficial, can act as a cathodic domain within galvanic microcells [36,37]—particularly when its distribution is heterogeneous or its morphology irregular.

Sample 4 (FA), with the lowest pearlite content (40.51%), showed the highest overall corrosion rate. The correlation diagram clarified this apparent contradiction by highlighting the influence of grain coarsening. Specifically, the ferrite D_max_ and ferrite area were both strongly and positively correlated with the corrosion indicators (CR and *I*_corr_), suggesting that excessive grain growth undermines corrosion resistance by reducing the grain boundary density and disrupting uniform film formation [37,38,39].

Sample 1 (N), with a moderate phase ratio and equiaxed grains, consistently aligned with favorable electrochemical indicators and mechanical properties. It demonstrated the highest *R*_p_ (167.3 Ω), the lowest *I*_corr_ (90.19 µA/cm^2^), and the most positive OCP (–0.5594 V), marking it as the condition with the best overall corrosion resistance.

The role of grain shape and size in governing corrosion behavior was further clarified through correlation with the EIS and PDP parameters. Grain circularity—both for ferrite and pearlite—showed a strong negative correlation with the corrosion rate and *I*_corr_, and a positive correlation with *R*_1_ and *R*_2_ [34,40]. This indicates that more equiaxed grains favor the development of more compact, continuous surface layers, which are less susceptible to localized degradation.

On the other hand, the grain area and maximum diameter were positively correlated with the corrosion indicators, indicating that coarsely grained microstructures are more vulnerable. Sample 6 (+FP), which had the most refined grains in terms of size, nonetheless exhibited low circularity and high morphological irregularity. These characteristics were strongly negatively correlated with *R*_p_ and *R*_1_, suggesting that extreme grain refinement, if not accompanied by geometric regularity, can result in poor corrosion performance [37,38,39].

Additionally, the correlation analysis revealed that hardness (HRB) was negatively correlated with ferrite grain size (D_max_, Equiv. Diameter, area) and positively correlated with ferrite circularity. These trends align with known metallurgical behavior, where finer and more uniform grains increase the dislocation density and hinder plastic deformation, thereby increasing hardness [41,42]. However, the relationship between hardness and corrosion resistance was not linear: Sample 6 exhibited the highest hardness but only moderate corrosion resistance due to poor grain morphology. This confirms that while grain refinement increases the mechanical strength, it must be balanced with morphological control to avoid compromising the corrosion performance [41].

The electrochemical data were interpreted synergistically to confirm the trends revealed by the correlation analysis.

Open circuit potential (OCP) showed a weak correlation with the corrosion rate, confirming that thermodynamic indicators alone are insufficient for predicting actual degradation [28].

Linear polarization resistance (LPR) values proved more predictive, with strong negative correlation with *I*_corr_ and CR and a positive correlation with *R*_1_. These trends were consistent with the microstructural findings; for example, Sample 1 exhibited both high *R*_p_ and a favorable grain structure.

Electrochemical impedance spectroscopy (EIS) reinforced these observations. Although *R_ct_* and *R_f_* are commonly associated with the charge transfer resistance at the metal interface and the resistance of the porous surface layer, in this study, their low values, combined with high CPEs and dispersion, indicate that the surface layer was porous, actively degrading, and lacked protective barrier properties [31,32,33]. Warburg impedance (*W*), representing diffusional effects, was highest in samples with irregular surface morphology, supporting the link between grain geometry and electrolyte interaction [40].

Potentiodynamic polarization (PDP) results offered kinetic validation. Both *I*_corr_ and the corrosion rate correlated strongly with coarseness and irregularity in grain morphology. The correlation diagram clearly highlighted that ferrite and pearlite D_max_, area, and perimeter were directly associated with higher corrosion activity, whereas equiaxed geometry and balanced phase distribution were consistently linked to reduced electrochemical reactivity [34,37,38].

## 5. Conclusions

In this study, the influence of different annealing treatments on the microstructure and corrosion behavior of 20MnCr5 steel in a 3.5% NaCl solution was investigated. A combination of microstructural analysis, hardness testing, and electrochemical techniques, including open circuit potential (OCP), linear polarization resistance (LPR), electrochemical impedance spectroscopy (EIS), and potentiodynamic polarization (PDP), was used to characterize each condition. To improve data interpretability, a correlation analysis was performed and visualized through a correlation diagram, enabling statistical evaluation of the relationships between grain characteristics, phase distribution, mechanical properties, and corrosion indicators.

The results show that the corrosion resistance of 20MnCr5 steel is not determined by a single parameter but by the interaction of grain size, morphology, and phase equilibrium. Key findings from each characterization method include the following:

In terms of microstructure, the normalized sample (N) exhibited refined equiaxed grains and a balanced ferrite-pearlite distribution, which was beneficial for corrosion resistance. In contrast, the fully annealed (FA) and transformation-annealed (+FP) samples had coarser and irregular grains, which negatively impacted the corrosion performance. The spheroidizing annealed sample (AC) exhibited carbide spheroidization beneficial for mechanical properties, but the corrosion performance varied due to uneven carbide distribution.

Phase analysis confirmed that the cementite distribution varied with cooling rates and was particularly favorable for the normalized (N) and spheroidizing annealed (AC) samples due to improved carbide morphology and uniformity.

Hardness measurements indicated that the transformation-annealed sample (+FP) had the highest hardness due to grain refinement, although its corrosion resistance was moderate due to irregular grain morphology. The fully annealed (FA) and spheroidizing annealed (AC) samples exhibited lower hardness values and varying corrosion behaviors, highlighting the need for balancing mechanical and corrosion properties.

Electrochemical tests demonstrated that the normalized sample (N) exhibited the best corrosion resistance, characterized by the lowest corrosion current density, highest polarization resistance, and most positive open circuit potential (OCP). The spheroidizing annealed (AC) and fully annealed (FA) samples showed higher corrosion rates and lower resistance, indicating susceptibility to localized corrosion. The transformation-annealed (+FP) sample exhibited moderate corrosion resistance due to refined but morphologically irregular grains.

These results emphasize the importance of precisely controlling the annealing parameters to adjust microstructural features and achieve an optimal balance between mechanical strength and corrosion resistance in 20MnCr5 steel, especially for components used in aggressive chloride environments. This knowledge is particularly important for designing components exposed to chlorides in marine or industrial applications, where the microstructure significantly influences the long-term performance.

## Figures and Tables

**Table 1:** Average chemical composition of the 20MnCr5 steel (balance Fe).

Chemical Composition, wt.%						
C	Si	Mn	P	S	Cr	Cu
0.218	0.257	1.01	0.00	0.0128	1.00	0.168

**Table 2:** Annealing heat treatments and parameters.

Sample No.	Heat Treatment	Temperature and Time/Cooling Process
1.	Normalizing (N)	890 °C for 1 h/Air cooling
2.	Stress relieving (+SR)	680 °C for 1 h/Slow furnace cooling
3.	Soft annealing (A)	700 °C for 1 h/Furnace cooling
4.	Full annealing (FA)	860 °C for 1 h, furnace cooling to 650 °C/Then air cooling
5.	Spheroidizing annealing (AC)	740 °C for 1 h, furnace cooling to 670 °C, pause 2 h, furnace cooling to 300 °C/Then air cooling
6.	Transformation annealing (+FP)	1000 °C for 15 min, quick cooling to 650 °C (1–2 s water dip, temperature check), 3 h holding/Then air cooling

**Table 3:** Phase ratio analyzed by Olympus Stream Essentials software.

Sample No.	Ratio of Pearlite (%)
1	66.82
2	48.82
3	64.91
4	40.51
5	71.53
6	54.89

**Table 4:** Results of the grain size analysis for the investigated 20MnCr5 steel samples.

White Grains/Ferrite Phase					
**Sample No.**	**D_max_**	**D_min_**	**Equiv. Diameter**	**Area**	**Circular**
1	22.91	13.98	15.73	232.68	0.60
2	21.42	12.92	14.70	193.36	0.62
3	22.79	13.34	15.41	218.83	0.60
4	41.49	25.87	28.96	888.27	0.58
5	22.40	13.83	15.65	226.13	0.61
6	23.17	10.87	12.74	150.67	0.42
**Dark Grains/Perlite Phase**					
**Sample No.**	**D_max_**	**D_min_**	**Equiv. Diameter**	**Area**	**Circular**
1	54.16	31.36	34.19	1067.55	0.47
2	55.25	34.91	36.96	1306.45	0.46
3	61.11	38.45	40.82	1572.21	0.47
4	35.85	22.36	24.92	550.53	0.57
5	56.39	36.05	39.11	1356.91	0.55
6	33.20	10.66	15.64	231.66	0.38

**Table 6:** OCP and *R*_p_ Values for all investigated 20MoCr5 steel samples in 3.5 wt.% NaCl solution.

Sample No.	OCP [V vs. Ag/AgCl]	*R*_p_ [Ω]
1	–0.5594	167.30
2	–0.5752	95.56
3	–0.5605	87.79
4	–0.5684	105.30
5	−0.5716	60.70
6	−0.5636	73.42

**Table 7:** Fitted EIS parameters obtained from equivalent circuit modeling of the impedance spectra for all samples.

Uzorak	*R*_s_ (Ω)	*R*_ct_ (Ω)	*R*_f_ (Ω)	CPE_dl_ (S·s^n1^)	*n*_1_	CPE_f_ (S·s^n2^)	*n*_2_	W (S·s^0.5^)
1	9.6447	61.4053	4.7482	0.00149	0.7966	0.001373	0.6794	0.2436
2	10.8153	54.0902	17.3466	0.003579	0.5642	0.001968	0.9280	0.2207
3	8.9033	45.8896	5.3159	0.000977	0.8694	0.000775	0.7460	0.2909
4	9.1419	46.3527	13.3335	0.009629	0.9121	0.003187	0.6411	0.3841
5	8.9165	29.8256	10.9576	0.001176	0.8979	0.003368	0.6523	0.5027
6	8.4175	40.7828	10.7763	0.001214	0.8894	0.002712	0.6242	0.3636

**Table 8:** Corrosion parameters for all investigated 20MoCr5 steel samples in 3.5 wt.% NaCl solution.

Sample No.	*E*_corr_ (mV)	*I*_corr_ (µA/cm^2^)	Corrosion Rate (mmpy)
1	−259.40	90.19	2.104
2	−254.70	297.80	3.662
3	−269.30	331.80	4.080
4	−255.40	622.40	7.654
5	−250.30	486.90	5.988
6	−258.10	266.60	3.279

## Data Availability

The data presented in this study are available on request from the corresponding author.

## Abbreviations

*A*_c1_	Lower critical temperature (start of austenitic transformation)
*A*_c3_	Upper critical temperature (complete austenitic transformation)
CPE	Constant phase element
CR	Corrosion rate
D_max_	Maximum grain diameter
D_min_	Minimum grain diameter
Equiv. Diameter	Equivalent grain diameter
HRB	Hardness Rockwell B
*I*_corr_	Corrosion current density
LPR	Linear polarization resistance
*R*_ct_	Charge transfer resistance
*R*_f_	Resistance of the porous corrosion surface layer
*R*_p_	Polarization resistance
*R*_s_	Solution resistance
